# Study of Purse-string Skin Closure Plus Negative-pressure Wound Therapy for Stoma Closure

**DOI:** 10.14789/jmj.JMJ22-0015-OA

**Published:** 2022-12-01

**Authors:** YU OKAZAWA, YUTAKA KOJIMA, KAZUHIRO TAKEHARA, SHOUKO NOJIRI, KOTA AMEMIYA, YUKI TSUCHIYA, Kumpei HONJO, Rina TAKAHASHI, MASAYA KAWAI, KIICHI SUGIMOTO, MAKOTO TAKAHASHI, KAZUHIRO SAKAMOTO

**Affiliations:** 1 Department of Coloproctological Surgery, Juntendo University Faculty of Medicine, Tokyo, Japan; 1 Department of Coloproctological Surgery, Juntendo University Faculty of Medicine, Tokyo, Japan; 2 Medical Technology Innovation Center Clinical Research and Trial Center Juntendo University, Tokyo, Japan; 2 Medical Technology Innovation Center Clinical Research and Trial Center Juntendo University, Tokyo, Japan; 3 Department of Surgery, Institute for Juntendo University Urayasu Hospital, Chiba, Japan; 3 Department of Surgery, Institute for Juntendo University Urayasu Hospital, Chiba, Japan

**Keywords:** stoma closure, purse-string skin closure, negative-pressure wound therapy

## Abstract

**Background:**

Although purse-string skin closure (PSC) is an effective method for stoma closure considering wound infection, the period for scarring will be prolonged. The aim of this study was to assess whether negative-pressure wound therapy (NPWT) can reduce the wound-scarring period for PSC after stoma closure.

**Methods:**

Patients who underwent stoma closure between January 2015 and August 2020 at our department were retrospectively assessed. Patients in the control group received only PSC, and patients in the NPWT group received both PSC and NPWT using the VAC^®^ or PICO^®^. The primary endpoint of this study was the short-term reduction ratio (RR). The RR is calculated by the length, width, and depth of the wound of the stoma closure site. The secondary endpoints were scarring period and wound-related complications such as surgical site infection, dermatitis, bleeding, enterocutaneous fistula, and ventral hernia.

**Results:**

Of the 53 patients included in this study, 21 had their stoma closed by PSC and 32 had their stoma closed by PSC plus NPWT. No significant differences were observed in patient characteristics or peri-operative states. The RR in the NPWT group was significantly smaller than that in the PSC group at 7 postoperative days (p=0.04). There was no difference in scarring period between the two groups (p=0.11).

The rates of postoperative wound-related complications were similar in the two groups (control group: 4 (19%), NPWT group: 7 (21.9%), p=1.0).

**Conclusions:**

Our study suggests that PSC plus NPWT might be more effective for wound healing after stoma closure than only PSC.

## Introduction

Recently, surgical techniques for lower rectal cancer have advanced and opportunities to select sphincter-preserving surgeries, such as super low anterior resection and intersphincteric resection, have increased^1)^, thus the frequency of diverting stoma construction has also increased^2)^.

Stoma closure is a relatively less-invasive surgery, but surgical site infection (SSI) is regarded as a problematic postoperative complication. SSI was reported in 10 to 45% as a complication after stoma closure^3-5)^. When SSI occurs, the patient's satisfaction with treatment decreases due to extension of the hospitalization period and increase in medical expenses.

As a method for preventing wound infection, purse-string skin closure (PSC) is available. In 1997, Banerjee et al. reported its tolerability and in 2002, Sutton et al. reported its preventative effects against SSI^6, 7)^. Although many reports mentioning its effectiveness have been published, PSC has problems. As it requires frequent washing, patients must wash the wound every day and the period for scarring is prolonged because the wound is open.

Negative-pressure wound therapy (NPWT) is a physical therapy that promotes wound healing through various effects. Wound sealing and the application of NPWT were reported to aid in wound contraction, increase wound blood flow, promote granulation tissue formation, reduce edema, and remove excess exudates and inactive tissues. Recently, there have been reports of NPWT being used for compromised wounds prophylactically with good results^8, 9)^. However, these reports focused on patients with primary linear closure of stoma wounds and did not evaluate healing. Uchino et al. conducted a randomized control trial (RCT) for patients with ulcerative colitis scheduled to undergo ileostomy closure. This study failed to demonstrate the efficacy of NPWT in significantly reducing the wound healing period^10)^. Following this study, Kim et al. are currently conducting an RCT on the effectiveness of NPWT for wound healing after stoma reversal^11)^.

In this study, to improve wound infection control and the cosmetic outcome after stoma closure, we examined the usefulness of the combination of PSC and NPWT.

## Materials and methods

We retrospectively reviewed the medical records of patients who underwent surgery for stoma closure at the Department of Coloproctological Surgery at Juntendo University Faculty of Medicine between January 2015 and August 2020. The patients were divided into 2 groups (control group: PSC alone, NPWT group: PSC plus NPWT) by surgeons. The study was approved by the research ethics committee of the Juntendo University Faculty of Medicine (Approval #IRB 20-191). Informed consent was received from all individual participants included in the study by the opt-out method.

The primary endpoint of this study was the short-term reduction ratio (RR). We measured the length, width, and depth of the wound of the stoma closure site on the 1st, 4^th^, and 7^th^ postoperative day (POD). The RR was calculated as follows:

$100-\frac{length\times width\times depth(mm) (4 or 7POD)}{length\times width\times depth(mm) (1POD)}\times 100$

Secondary endpoints were postoperative wound- related complications (POCs) such as surgical site infection (SSI), dermatitis, bleeding, enterocutaneous fistula, and ventral hernia. We defined events over Clavien-Dindo classification (CD) grade 2 as POCs. SSI was defined by the current Center for Disease Control (CDC) guidelines^12)^. The follow-up periods for enterocutaneous fistula and ventral hernia were within the observation periods, and those for others were 30 days after operation.

Outpatient follow-up after discharge was generally scheduled 3 or 4 weeks after discharge, but the schedule thereafter was at the discretion of the outpatient doctor. Whether the stoma reversal wound scarred completely was judged by the medical records.

### Surgical technique

Stoma reversal was performed using standard techniques with the patient under general anesthesia, and the bowel anastomosis was either stapled or hand-sewn at the discretion of the surgeon. After fascia closure, skin closure was performed with the subcuticular purse-string method using absorbable monofilament suture material (1 Maxon^TM^; Covidien, Dublin, Ireland or No.1 PDS plus^®^; Ethicon, Cincinnati, Ohio., USA). The wound was slightly opened as a drain hole to enable insertion of the tip of the 5-mL syringe to unify its size.

In the control group, patients continued washing with saline or water, and dressings were changed once a day.

In the NPWT group, NPWT was applied 24 hours after surgery after the confirmation of no postoperative bleeding using the V.A.C^®^ system (Kinetics Concepts Inc. [KCI], an Acelity Company, San Antonio, Texas, USA) or PICO^®^ system (Smith & Nephew Healthcare, Hull, UK). The suction pressure of the VAC^®^ system was set 120mmHg, and PICO^®^ was 80mmHg. It was reattached every three days after surgery and removed on 7POD.

### Statistical analysis

Categorical variables were compared using the χ2 test for multiple outcomes, Fisher's exact test for dichotomous outcomes involving small samples, Student's t test for continuous variables with normal distribution, or the Mann–Whitney nonparametric test for non-normal distributions. The level of significance was set at p<0.05. Analyses were performed using JMP software, version 14, from SAS Institute Inc. (Cary, NC).

## Results

**Figure 1:**
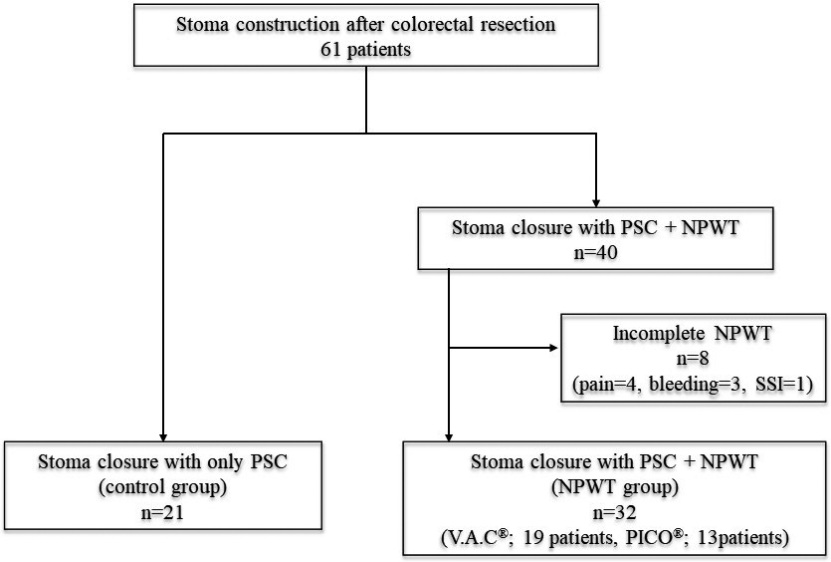
The patient selection flowchart PSC: purse-string skin closure NPWT: negative-pressure wound therapy

Between January 2015 and June 2020, 61 patients underwent stoma construction after colorectal surgery. Twenty-one patients received only PSC (control group), and 40 received PSC and NPWT (NPWT group). Eight patients who did not fully receive NPWT were excluded. The causes of complications were pain due to suction pressure in 4 patients, bleeding in 3, and intraabdominal abscess in 1. In total, 53 patients were included in the analysis, 21 of whom had their stoma closed by PSC and 32 who had their stoma closed by PSC plus NPWT. The V.A.C^®^ system was used for 19 patients and the PICO^®^ system was used for 13 (Figure 1).

**Figure 2:**
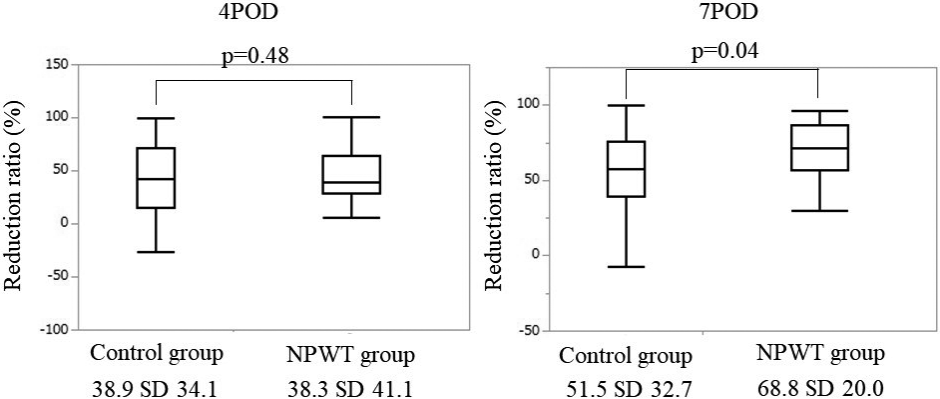
Comparison analysis for reduction ratio between closure methods POD: postoperative days NPWT: negative-pressure wound therapy

**Figure 3:**
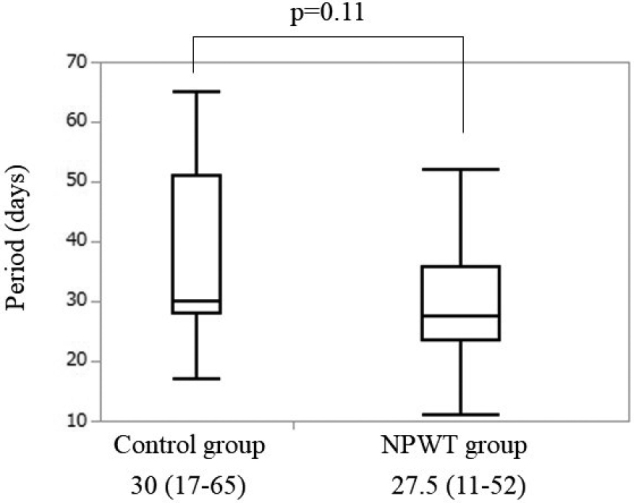
Comparison analysis for complete scarring period between closure methods NPWT: negative-pressure wound therapy

No significant differences were observed in sex, age, body mass index, neoadjuvant chemotherapy, diabetes, corticosteroids, preoperative albumin level, type of stoma, stoma holding period, duration of surgery, operative blood loss or follow-up period between the two groups (Table 1). The RR of patients in the control group was 38.9±34.1% (4POD) and 51.5±32.7% (7POD), and that in the NPWT group was 38.3±41.1% (4POD) and 68.8± 20.0% (7POD) (Figure 2). The RR in the NPWT group was significantly smaller than that in the control group at 7POD (p=0.04), although there was no significant difference between the two groups at 4POD (p=0.48). No significant difference was observed between the two groups in the scarring period (p=0.11). Patients in the control group and NPWT group required 30 (17-65) and 27.5 (11-52) days for complete wound scarring, respectively (Figure 3).

**Table 1:** Patient characteristics

	Control (N＝21)	NPWT (N＝32)	P-value
Sex (Man / Woman)	17 / 4	18 / 14	0.06
Mean age (year)±SD	61.5±11.0	62.3±13.9	0.58
Mean body mass index (kg/m²)±SD	22.0±2.0	21.6±3.0	0.31
Chemotherapy, n (%)	9 (42.9%)	11 (34.4%)	0.53
Diabetes, n (%)	3 (14.3%)	5 (15.6%)	1.0
Corticosteroid, n (%)	0 (0%)	1 (3.1%)	1.0
Mean preoperative albumin level (g/ml) ±SD	4.1±0.32	4.1±0.43	0.30
Type of stoma (ileostomy / colostomy)	20 / 1	27 / 5	0.38
Median stoma holding period (days)	238 (34-569)	175 (19-814)	0.20
Median duration of surgery (min)	96 (64-152)	87 (56-424)	0.48
Median operative blood loss (ml)	15.0 (3-80)	11.5 (3-370)	0.32
Median hospital stay (days)	8.0 (7-35)	8.0 (7-22)	0.66
Median following period (days)	639 (7-1758)	862 (8-1337)	0.74
Wound size at 1POD (length×width×depth(mm))	4500 (1700-16500)	7050 (1000-22500)	0.18
Wound size at 4POD (length×width×depth(mm))	3150 (15-14212)	3825 (245-28512)	0.23
Wound size at 7POD (length×width×depth(mm))	2366 (15-9856)	1650 (180-9000)	0.45

Postoperative wound-related complications were observed in 4 (19%) patients in the control group and in 7 (21.9%) patients in the NPWT group (p=1.0). Incisional SSI was confirmed in 1 patient in each group. Dermatitis was confirmed in 2 patients in the control group and in 1 patient in the NPWT group. Bleeding was confirmed in 2 patients in the NPWT group. All of these events were CD grade 2 or less. Enterocutaneous fistula was not noted in either group. Ventral hernias were confirmed in 1 patient in the control group and 3 patients in the NPWT group (Table 2).

**Table 2:** Postoperative wound-related complications

	Control (N＝21)	NPWT (N＝32)	P-value
All complications, n (%)	4 (19.0)	7 (21.9)	1.0
Early complications			
SSI Incisional, n (%)	1 (4.8)	1 (3.1)	
Organ / Space, n (%)	0 (0)	0 (0)	
Dermatitis, n (%)	2 (9.5)	1 (3.1)	
Bleeding, n (%)	0 (0)	2 (6.3)	
Late complications			
Enterocutaneous fistula, n (%)	0 (0)	0 (0)	
Ventral hernia, n (%)	1 (4.8)	3 (9.4)	

## Discussion

Stoma closure is generally considered minor surgery, although the rate of complications is high at 20 to 40%. Stoma closure is classified as a class 3 contaminated wound, which has a high risk of SSI. SSI is one of the most common complications in the postoperative treatment of contaminated wounds. Many surgical techniques have aimed at improving outcomes by reducing the rate of SSI. PSC was first introduced by Banerjee et al. to reduce SSI after stoma closure^6)^. In 2002, Sutton et al. reported good results of a 0% SSI rate after PSC^7)^. In 2007, Haku et al. compared PSC with simple closure, and reported an SSI rate of 27% by simple closure and 0% by PSC. In addition, in 2009 and 2010, Milanchi et al. and Marquez et al., respectively, reported that the SSI rate after simple closure was 40% and 18%, but 0% by PSC^13, 14)^. In 2014, Muhammad et al. reported a comparative study by meta-analysis of simple closure and PSC for stoma closure. PSC significantly reduced the SSI compared with simple closure^15)^. Although the rate of SSI decreased, the wound-scarring period for PSC after stoma closure is longer than that required for the primary closure method.

NPWT is applied for many types of wounds in order to reduce the incidence of SSI and shorten the healing period^16-18)^. Previous reports considered the use of NPWT for open abdominal management or wound infection; however, the prophylactic use of NPWT is still not considered important in digestive surgery. There is only one report of NPWT for closure of colostomy, in which Uchino et al. used NPWT for inflammatory bowel disease patients in 2016^10)^. According to that report, the incidence of incisional SSI was similar between the groups (p=0.76). The mean duration of complete wound healing was 37.6±11.7 days by PSC alone and 33.5± 10.0 days by PSC+NPWT. Although no adverse effects were observed in this series, the efficacy of PSC+NPWT was not confirmed. Recently, two reports regarding NPWT for ileostomy closure in colorectal cancer were published. Wierdak et al. reported a comparative study of primary wound closure with or without NPWT. Patients in the NPWT group had a significantly lower incidence of SSI (5.71% vs. 22.2%; p=0.046) and significantly shorter complete wound healing time (median 7[7-7] days vs. 7[7-15] days p=0.03)^19)^. Okuya et al. the effectiveness of preventive NPWT for SSI development after ileostomy closure^20)^. In this report, none of the patients were diagnosed with SSI, seroma or hematoma. NPWT-related complications, such as dermatitis and wound pain, were noted in two and one patient each.

In our study, incisional SSI developed in 4.8% patients with NPWT and in 3.1% patients without NPWT. The incidence of incisional SSI was similar between the groups, consistent with previous reports. The median RR was 51.5±32.7% in the PSC alone group and 68.8±20.0% in the PSC+NPWT group at 7POD. The median scarring period was 30 (17-65) days in the PSC alone group and 27.5 (11-52) days in the PSC+NPWT group. A significant difference was observed in the RR (p=0.04), but not in the scarring period (p=0.11). One reason for the lack of a significant difference in the scarring period was that the small wound size precluded the effects of NPWT. If the wound is larger, there may be a more significant difference in the RR and scarring period.

In our study, some NPWT-related problems were observed. NPWT was unable to be performed completely due to pain (4 patients), bleeding (3 patients) or SSI (1 patient) (Figure 3). The V.A.C^®^ system was used for 5 of 8 patients with these NPWT-related problems. As the V.A.C^®^ system has stronger suction pressure than the PICO^®^ system, complications, such as pain and bleeding, may have been due to the suction pressure of NPWT. There are few reports on the safety of NPWT for PSC; therefore, further studies are required.

There are several limitations to this study. First, it was a single-institutional superior trial. As the number of patients was insufficient to examine superiority, multi-institutional clinical trials are expected in the future. Second, both the V.A.C^®^ system and PICO^®^ system were used as NPWT in this study. In general, the suction pressure of V.A.C^®^ is 120 mmHg and that of PICO^®^ is 80 mmHg, thus the suction pressure of V.A.C^®^ is stronger. This inconsistency may have affected the outcome. Third, we may have incorrectly assessed the scarring period. As the schedule of outpatient visits after discharge was not set in advance, there may have been a difference in scarring period depending on the day of outpatient visit. Fourth, this study did not evaluate the cost effectiveness of prophylactic NPWT or patient satisfaction (cosmetic outcome and difficulty of wound care). These factors should be assessed in future studies. We are awaiting the results of the SR-PICO study^11)^.

## Conclusion

In this study, PSC+NPWT was suggested to might be effective for early wound healing of stoma closure. NPWT-related complications are infrequent, but care for pain and bleeding is needed when using NPWT.

## Funding

The authors received no financial support for the research.

## Author contributions

YK, KT, KA, YT, KH, RT, MK, KS and MT made substantial contributions to conception and design, acquisition of data. SN analysis and interpretation of data and statistical analysis. KS made contributions to drafting the article; and final approval of the version to be published. All authors read and approved the final manuscript.

## Conflicts of interest statement

The Authors declare that there are no conflicts of interest.
